# Geographical distribution and phoretic associations of the viviparous nematode *Tokorhabditis atripennis* with *Onthophagus* dung beetles in Japan

**DOI:** 10.2478/jofnem-2024-0013

**Published:** 2024-04-25

**Authors:** Yuya Ikeda, Yuto Koike, Ryoji Shinya, Koichi Hasegawa

**Affiliations:** School of Agriculture, Meiji University, Kawasaki, Kanagawa, Japan; Department of Environmental Biology, College of Bioscience & Biotechnology, Chubu University, Kasugai, Japan

**Keywords:** insect-nematode interaction, distribution, viviparity, dung beetle, ecology

## Abstract

Viviparity is generally considered to be rare in animals. In nematodes, only six species of Rhabditida are viviparous. Five of these species have been identified in association with *Onthophagus* dung beetles, with *Tokorhabditis atripennis* being repeatedly isolated from the dung beetle *Onthophagus atripennis* in Japan. *T. atripennis* is easy to culture in a laboratory setting, and its host, *O. atripennis*, is distributed all over Japan. Therefore, *T. atripennis* is an ideal candidate for ecological and evolutionary studies on viviparity. However, the extent of their distribution and relationship with dung beetles, as well as habitats, remain unclear. In the present study, we conducted field surveys and successfully isolated 27 strains of viviparous nematodes associated with tunneler dung beetles from various regions of Japan, all of which were identified as *T. atripennis*. *T. atripennis* exhibited a strong association with *Onthophagus* dung beetles, especially *O. apicetinctus* and *O. atripennis*. And it was predominantly found in specific anatomical locations on the beetle bodies, such as the ‘groove between pronotum and elytron’ and the ‘back of the wings’. Our findings suggest that *Onthophagus* species are the primary hosts for *T. atripennis*, and *T. atripennis* exhibits a close relationship with the living environments of tunneler beetles. This association may play a significant role in the evolution of viviparity in nematodes.

Throughout their evolutionary history, animals have developed diverse reproductive strategies, adapting to a wide range of ecological systems. Among animals, viviparity stands out as one of the most prevalent reproductive modes, primarily employed as a strategy to increase the body size of offspring before delivery rather than their quantity. Viviparity is an incubation mode in which embryonic development occurs within the reproductive system (ovary or sexual duct), body cavity (coelom, pseudocoel, or hemocoel), parental tissues, or tissue-like layers (parenchyma, mesohyl, or mesoglea) with nutrient supply, resulting in live births (Ostrovsky et al., 2016). It has evolved independently at least 160 times (Blackburn, 1999), suggesting that certain environmental factors influence the evolution of viviparous traits in these animals. However, empirical research demonstrating its specific role and the driving forces behind its evolution remains largely lacking in many animal species.

Although nematodes are generally oviparous, females of some nematode species retain fertilized eggs and are described as viviparous. Viviparity in the Rhabditida is typically facultative, either manifesting as *endotokia matricida* (“bagging”; also “aparity,” *sensu* Sudhaus 1976) or as live birth of young that hatch from rigid eggs in utero (Johnigk and Ehlers, 1999; Chen and Caswell-Chen, 2004; Vigne et al., 2021). However, only six species within the Rhabditida appear to meet the definition of obligate viviparity, which obligately retains fertilized eggs with embryonic growth and nutritional provision (Herrmann et al., 2013; Kanzaki et al., 2017, 2021; Ragsdale et al., 2022; Yamashita et al., 2023). Of these, five have been found to be associated with *Onthophagus* dung beetles, with the exception of *Tokorhabditis tufae*, which was discovered in the alkaline, hypersaline, and arsenic-rich environment of Mono Lake, California (Shih et al., 2019). One such species, *Tokorhabditis atripennis* was discovered in association with the dung beetle *Onthophagus atripennis* (Ragsdale et al., 2022). *Onthophagus* dung beetles engage in tunneling beneath animal dung, crafting brood balls from the dung that serve as nourishment and nesting sites for their larvae, a behavior indicative of tunnelers (Bornemissza, 1969; Bornemissza, 1976; Cambefort and Hanski, 1991). This suggests that the viviparous nematode *T. atripennis* is a dung beetle-phoretic species dwelling within fecal environments, including brood balls, and that its reproductive mode is an adaptation to these specialized habitats.

Given that *T. atripennis* is readily found in association with *Onthophagus* dung beetles in the field and can be cultured in the laboratory, the nematode-dung beetle system is an excellent model for studying the evolution of animal reproduction. However, because of the lack of a comprehensive sampling survey of *T. atripennis*, the following essential questions remain unanswered: (1) the preferred dung beetle taxon for *T. atripennis* as a host, (2) whether *T. atripennis* exhibits phoretic behavior with dung beetles (with the potential for parasitism or accidental association), and (3) the geographical range of its distribution. Although viviparity in *T. atripennis* appears to be an adaptation to dung beetle habitats, the lack of ecological information on this nematode makes it challenging to uncover the potential factors behind its viviparous reproductive strategy.

In this study, we aimed to investigate the relationship between *T. atripennis* and dung beetles and to gather ecological information regarding their distribution. We conducted fieldwork and collected data on 615 dung beetles from 12 Japanese prefectures. Subsequently, we performed dissections and investigated the following aspects: 1) The rate of association between *T. atripennis* and each dung beetle species; 2) Specific anatomical locations within dung beetle bodies where nematodes were found; 3) Regional variations in the prevalence of *T. atripennis* associations with dung beetles across Japan.

## Materials and Methods

### Dung beetle collection and dissection

To isolate *T. atripennis*, we collected dung beetles using various methods, including pit-hole traps, direct capture from within or beneath animal droppings, excavation of underground nests, and nocturnal searches among insects attracted to artificial light. For the pit-hole traps, we dug the soil surface and placed paper cups at a volume of 105 ml at ground level. Rotted fish meal was used as an attractant for dung beetles. Pit-hole traps were set up at four distinct locations: Kasugai campus of Chubu University, Kasugai, Japan (Coordinates, 35° 16′ 31.8″ N, 137° 00′ 58.6″ E; Date, 2016.6.5, 7.5, 11.5, 2017.5.8, 5.10, 5.26, 2020.11. 16 to 28), Ena Campus of Chubu University, Ena, Japan (Coordinates, 35° 25′ 45.6″ N, 137° 21′ 15.2″ E; Date, 2016.5.22), Ikuta Campus of Meiji University, Kawasaki, Japan (Coordinates, 35° 36′ 39.8″ N, 139° 32′ 55.8″ E; Date, 2021.5.14, 8.2 to 4, 9.16) and at the Meiji University Kurokawa Field Science Center, Kawasaki, Japan (Coordinates, 35° 36′ 31.5″ N, 139° 27′ 20.8″ E; Date, 2021.9.30, 10.20, 10.21). Details of the dung beetles collected using methods other than pit-hole traps are described in Table 1.

The collected beetles were individually stored in plastic cases with humid tissue paper until dissection. Before dissection, each beetle’s viability was confirmed, and they were observed under a stereo microscope for species and sex identification following the illustration reference, “Scarab beetles of Japan” (Kawai et al., 2008). Most beetles were able to survive for up to one week using this sampling method. However, if any beetles died, they were excluded from further study.

**Figure 1:**
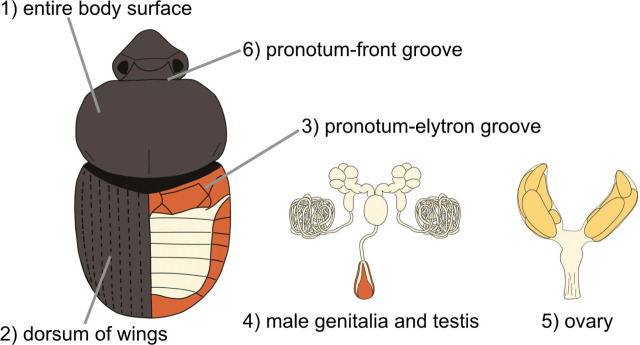
The body parts of beetles examined the number of *Tokorhabditis atripennis* associated.

The dung beetles were dissected to verify the presence or absence of phoretically associated nematodes. We identified six distinct body parts of the nematode: 1) the entire body surface, 2) the dorsum of the wings, 3) the pronotum-elytron groove, 4) the male genitalia and testis, 5) ovary, and 6) the pronotum-front groove and the anterior regions for locating the phoretic nematodes (Fig. 1). We carefully rinsed the surface of the dung beetles with ion exchange water (IEW) on a Syracuse watch glass and looked for nematodes under a dissecting microscope. Subsequently, we dissected the beetle elytron and examined the dorsal area as well as the groove between the pronotum and the elytron in the presence of nematodes. Next, we removed and examined the male genitalia, testes, and ovaries for the presence of nematodes. Then, we detached the heads of dung beetles from the pronotum and observed the presence of nematodes.

**Table 1:** The number of beetles collected for each attractant and collection site, with the exception of pit-hole traps.

**Attractant**	**Date**	**Collecting site**	**Beetles number**
Extensive raising deer dropping	2017.5.27	Araike, Nara Park, Nara	35
		Kasugano Enchi, Nara Park, Nara	26
	2017.5.28	Araike, Nara Park, Nara	8
		Asajigahara, Nara Park, Nara	19
	2018.7.24	Araike, Nara Park, Nara	19
		Asajigahara, Nara Park, Nara	74
Extensive raising cow dropping	2017.6.24	Chausuyama, Kitashitara, Aichi	47
Wild horse dropping	2017.7.2	Toino misaki, Kushima, Miyazaki	8
Domestic cow dropping	2017.7.4	Yakushima, Kagoshima	15
Dog or Cat dropping	2017.7.7	Ishigaki, Okinawa	7
	2018.12.1	Matsuo Park, Naha, Okinawa	1
		Chuo Park, Naha, Okinawa	2
		Midorigaoka Park, Naha, Okinawa	14
	2018.12.3	Tonoshiro Park, Ishigaki, Okinawa	4
	2018.12.2	Tonoshiro Park, Ishigaki, Okinawa	25
		Tonoshiro Park, Ishigaki, Okinawa	20
		Shogyo ji, Ishigaki, Okinawa	3
Hand catch	2017.8.28	Kurama, Kyoto	2
	2021.9.16	Meiji Univ, Kawasaki, Kanagawa	2
Wild deer dropping	2017.9.26	Kamaishi, Iwate	6
	2017.10.16	Kagoshima Univ, Takakuma, Kagoshima	2
	2017.11.1	Shiriyazaki, Aomori	17
	2018.5.11	Morioka, Iwate	13
	2018.8.29	Asahisashi, Kamaishi, Iwate	39
		Ogawa, Kamaishi, Iwate	29
Domestic sheep dropping	2018.8.22	Nakasatsunai, Hokkaido	5
Vending machine light	2018.8.28	Morioka, Iwate	2
Extensive raising horse dropping	2018.9.4	Aso, Kumamoto	35
Streetlight	2021.6.25	Atsuma, Hokkaido	4
	2021.7.17	Uehara, Taketomi, Yaeyama, Okinawa	8

Total			491

**Figure 2:**
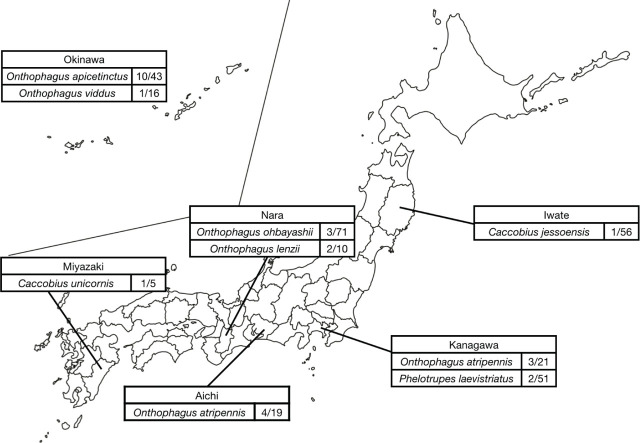
Geographical distribution of *Tokorhabditis atripennis* in Japan. The numbers in the figure are the proportions of test beetles that were associated with *T. atripennis*.

**Table 2:** Information on all *Tokorhabditis atripennis* strains obtained in this study.

**Strain**	**Date**	**Collection Site**	**Host**	**Beetles’ body site**
KHA602	2016.6.5	Chubu Univ, Kasugai, Aichi	*O. atripennis*	-
KHA603	2016.6.5	Chubu Univ, Kasugai, Aichi	*O. atripennis*	-
KHA604	2016.6.5	Chubu Univ, Kasugai, Aichi	*O. atripennis*	-
KHA605	2017.5.10	Chubu Univ, Kasugai, Aichi	*O. atripennis*	1), 2)
KHA606	2017.5.27	Kasugano Enchi, Nara Park, Nara	*O. lenzii*	2), 4)
KHA607	2017.5.28	Asajigahara, Nara Park, Nara	*O. ohbayashii*	3)
KHA608	2017.5.28	Asajigahara, Nara Park, Nara	*O. lenzii*	1), 2), 3)
KHA612	2017.7.2	Toino misaki, Kushima, Miyazaki	*C. unicornis*	2)
KHA611	2017.7.7	Ishigaki, Okinawa	*O. apicetinctus*	2)
KHA609	2017.7.7	Ishigaki, Okinawa	*O. apicetinctus*	2)
KHA610	2017.7.7	Ishigaki, Okinawa	*O. viduus*	3)
KHA613	2018.5.11	Morioka, Iwate	*C. jessoensis*	3)
KHA614	2018.7.24	Asajigahara, Nara Park, Nara	*O. ohbayashii*	2)
KHA615	2018.7.24	Asajigahara, Nara Park, Nara	*O. ohbayashii*	3)
KHA616	2018.12.3	Tonoshiro Park, Ishigaki, Okinawa	*O. apicetinctus*	2)
KHA619	2018.12.2	Shinsakae Park, Ishigaki, Okinawa	*O. apicetinctus*	3)
KHA620	2018.12.2	Tonoshiro Park, Ishigaki, Okinawa	*O. apicetinctus*	3)
KHA617	2018.12.2	Tonoshiro Park, Ishigaki, Okinawa	*O. apicetinctus*	3)
KHA621	2018.12.2	Tonoshiro Park, Ishigaki, Okinawa	*O. apicetinctus*	3)
KHA622	2018.12.2	Tonoshiro Park, Ishigaki, Okinawa	*O. apicetinctus*	3)
KHA623	2018.12.2	Tonoshiro Park, Ishigaki, Okinawa	*O. apicetinctus*	2)
KHA618	2018.12.2	Tonoshiro Park, Ishigaki, Okinawa	*O. apicetinctus*	3)
SHR9	2021.5.14	Meiji Univ, Kawasaki, Kanagawa	*O. atripennis*	2)
SHR12	2021.8.3	Meiji Univ, Kawasaki, Kanagawa	*P. laevistriatus*	1)
SHR21	2021.8.4	Meiji Univ, Kawasaki, Kanagawa	*O. atripennis*	3)
SHR24	2021.8.4	Meiji Univ, Kawasaki, Kanagawa	*P. laevistriatus*	6)
SHR16	2021.9.30	Kurokawa Field Science Center, Kawasaki, Kanagawa	*O. atripennis*	2), 3)

### Nematode isolation

Once nematodes were found during the dissection of dung beetles, we transferred them onto nematode growth medium (NGM) (Brenner, 1974), as well as onto the NGM + dog food medium (DFM: 20 g crushed dog food, 4 g agar and IEW were poured until the total volume became 200 ml, autoclaved and solidified) seeded with *E. coli* OP50 and incubated them at 25 °C (Hara et al., 1981; Ogura and Mamiya, 1989). Two to three days after being transferred to NGM or DFM plates, most free-living nematodes reached the adult stage, allowing us to distinguish their reproductive modes as either oviparous or viviparous. Viviparous nematodes were sterilized using a 10% SDS solution and established as laboratory strains, except for the strains of SHR that were just washed in IEW. Although oviparous nematodes were collected in this study, they were not identified as strains.

### Molecular profiles and phylogeny

Genomic DNA were extracted from nematodes using the Qiagen DNeasy Blood and Tissue Kit (Qiagen, USA) or Direct PCR Lysis Reagent (Viagen Biotech, USA). The D1/D4 or D2/D3 extension segments of the 28S ribosomal RNA gene (LSU) (Kanzaki et al., 2021; Nunn, 1992) and a partial fragment of the 18S ribosomal RNA gene (SSU) (Floyd et al., 2005; Carta and Li., 2018) were amplified and sequenced using universal primers (Table S1). The obtained DNA fragments were purified from agarose gels with NucleoSpin^®^ Gel and PCR Clean-up (Macherey-Nagel, Germany) or FastGene Gel/PCRExtraction kit (NIPPON Genetics Co., Ltd, Japan). Samples were submitted to Hokkaido System Science Co. (Sapporo, Japan) or Macrogen Japan Corp. for sequencing from both strands using the same PCR primers. The sequences obtained were confirmed and edited manually using a Serial Cloner (ver. 2-6-1) and identified to the species level based on the results of the Basic Local Alignment Search Tool (BLAST) analysis. Isolated nematodes were considered potentially distinct species if they exhibited <99% sequence similarity to their nearest neighbors. Sequence data were deposited in the NCBI for Biotechnology Information GenBank database.

### Statistical analysis

Significant differences in the percentages of dung beetles associated with viviparous nematodes among regions, beetle genera, and species were determined. First, a Fisher’s exact test was performed using the R package in Jupyter Lab. When significant differences were detected by the Fisher’s exact test, Tukey’s WSD was performed for multiple comparisons at *P < 0.05*.

**Table 3:** Percentage of dung beetles associated with *Tokorhabditis atripennis*.

**Host**		**% beetles associated with nematodes to total examined beetles (no. associated/no. examined)**	
**Genus**	**Species**		
*Aphodius*		0.00 (0/103)	
*Ataenius*		0.00 (0/8)	
*Copris*		0.00 (0/25)	
*Caccobius*		1.69 (2/118)	
	*Caccobius jessoensis*		1.22 (1/82) ^a^
	*Caccobius unicornis*		3.13 (1/32) ^ab^
*Onthophagus*		8.33 (23/276)	
	*Onthophagus apicetinctus*		23.3 (10/43) ^bc^
	*Onthophagus atripennis*		14.9 (7/47) ^bc^
	*Onthophagus lenzii*		6.67 (2/30) ^abc^
	*Onthophagus ohbayashii*		4.11 (3/73) ^abc^
	*Onthophagus viduus*		5.26 (1/19) ^abc^
*Phelotrupes*		2.35 (2/85)	
	*Phelotrupes laevistriatus*		2.47 (2/81) ^a^

Total		4.07 (27/615)	

Different letters indicate significance differences (*P* < 0.05, Tukey WSD). Species names are written only when the beetles were associated.

## Results

### All isolated free-living viviparous nematodes were *T. atripennis*

A total of 615 dung beetles were collected from 12 prefectures and classified into 30 species across six genera (Table S2). Most of the nematodes associated with dung beetles were free-living nematodes with oviparous reproduction (data not shown). This was evident when eggs were laid on plates seeded with *E. coli* OP50 after reaching adulthood. However, a subset of the isolated nematodes did not lay eggs, but released larvae, leading us to classify them as exhibiting viviparous reproduction.

All isolated viviparous nematodes were successfully cultured on plates, genomic DNA was isolated, and sequences of the LSU and SSU genes were obtained. In total, 27 viviparous nematode strains were obtained from individual dung beetles of eight species found in six prefectures (Fig. 2; Table 2). Upon comparing their LSU and SSU gene sequences, it was evident that all genes in the 27 strains were identical to those of *T. atripennis.* The NCBI accession numbers assigned to the gene sequences of each strain are shown in Table S3.

### Prevalent association of *T. atripennis* with *Onthophagus* beetles

Most dung beetles associated with *T. atripennis* were members of the genus *Onthophagus* (23/276). A few beetles belonging to the genera *Caccobius* (2/118) and *Phelotrupes* (2/85) were also associated with *T. atripennis*. The association rate differed significantly between *Onthophagus* and *Phelotrupes* (Tukey’s WSD, q=3.3145, wsd=0.05945). *T. atripennis* was not isolated from the beetles belonging to the genera *A*p*hodius* (n=103), *Ataenius* (n=8), or *Copris* (n=25) (Table 3). Across all beetle species, approximately 70% of *T. atripennis* detected in this study were isolated from two species, *O. apicetinctus* and *O. atripennis*, both of which belong the genus *Onthophagus*. When multiple comparisons were performed (Tukey’s WSD, q=4.286, *P* < 0.05), significant differences in association rates were observed among the three cohorts: *Caccobius jessoensis* and *Onthophagus apicetinctus* (wsd=0.1420), *Phelotrupes laevistriatus* and *O. apicetinctus* (wsd=0.1530), and *O. atripennis* and *C. jessoensis* (wsd=0.1139). Although there was no significant association between *Onthophagus* species and viviparous nematodes, the rates of two *Onthophagus* species, *O. atripennis* and *O. apicetinctus*, were notably elevated compared to other *Onthophagus* species (Table 3). This suggests a preference for viviparous nematodes with specific *Onthophagus* species as hosts.

### Localization of *T. atripennis* on specific body regions of the beetles

During the dissection of dung beetles, we investigated the nematode-associated sites on the beetles’ bodies by categorizing them into six parts:1) entire body surface, 2) dorsum of the wings, 3) pronotum-elytron groove, 4) male genitalia and testis, 5) ovary, and 6) pronotum-front groove. In general, *T. atripennis* was primarily associated with 2) the dorsum of the wings and 3) the pronotum-elytron groove, with no instances of *T. atripennis* detected in 5) the ovaries (Table 4). In the *Onthophagus* genus, *T. atripennis* was observed on 2) the dorsum of the wings (10/24) and 3) the pronotum-elytron groove (11/24). Nematodes were detected on 1) the entire body surface (2/24) and 4) the male genitalia and testis. In *Caccobius* beetles, *T. atripennis* has been isolated from 2) dorsum of the wings (1/3), 3) pronotum-elytron groove (1/3), and 4) male genitalia and testes (1/3). In *Phelotrupes* beetles, *T. atripennis* was isolated from 1) the entire body surface (1/2) and 6) the groove between the pronotum and front (1/2).

**Table 4:** The number of *Tokorhabditis atripennis* isolated from each body part of dung beetles.

**Host**		**% associated (no. associated body part/no. examined)**					

**Genus**	**Species**	**(1)**	**(2)**	**(3)**	**(4)**	**(5)**	**(6)**
*Caccobius*		0.00 (0/3)	33.3 (1/3)	33.3 (1/3)	33.3 (1/3)	0.00 (0/3)	0.00 (0/3)
	*Caccobius jessoensis*	0.00 (0/2)	0.00 (0/2)	50.0 (1/2)	50.0 (1/2)	0.00 (0/2)	0.00 (0/2)
	*Caccobius unicornis*	0.00 (0/1)	100 (1/1)	0.00 (0/1)	0.00 (0/1)	0.00 (0/1)	0.00 (0/1)
*Onthophagus*		8.33 (2/24)	41.7 (10/24)	45.8 (11/24)	4.17 (1/24)	0.00 (0/24)	0.00 (0/24)
	*Onthophagus apicetinctus*	0.00 (0/10)	40.0 (4/10)	60.0 (6/10)	0.00 (0/10)	0.00 (0/10)	0.00 (0/10)
	*Onthophagus atripennis*	16.7 (1/6)	50.0 (3/6)	33.3 (2/6)	0.00 (0/6)	0.00 (0/6)	0.00 (0/6)
	*Onthophagus lenzii*	20.0 (1/5)	40.0 (2/5)	20.0 (1/5)	20.0 (1/5)	0.00 (0/5)	0.00 (0/5)
	*Onthophagus ohbayashii*	0.00 (0/2)	50.0 (1/2)	50.0 (1/2)	0.00 (0/2)	0.00 (0/2)	0.00 (0/2)
	*Onthophagus viduus*	0.00 (0/1)	0.00 (0/1)	100 (1/1)	0.00 (0/1)	0.00 (0/1)	0.00 (0/1)
*Phelotrupes*		50.0 (1/2)	0.00 (0/2)	0.00 (0/2)	0.00 (0/2)	0.00 (0/2)	50.0 (1/2)
	*Phelotrupes laevistriatus*	50.0 (1/2)	0.00 (0/2)	0.00 (0/2)	0.00 (0/2)	0.00 (0/2)	50.0 (1/2)

Total		10.3 (3/29)	37.9 (11/29)	41.4 (12/29)	6.90 (2/29)	0.00 (0/29)	3.45 (1/29)

The numbers in this table mean (1) entire body surface, (2) back of the wings, (3) pronotum-elytron groove, (4) male genitalia and testis, (5) ovary, and (6) pronotum-front groove.

### Geographical distribution of *T. atripennis* across Japan

In terms of individual prefectures, the highest association rate of *T. atripennis* was observed in Okinawa (11/84), followed by Miyazaki (1/8) and Kanagawa (5/90). The rates in the other three prefectures were all below 5%, as shown in Table 5. Among *Onthophagus* species, *T. atripennis* was detected in four prefectures: Aichi (4/45), Kanagawa (3/39), Nara (5/95), and Okinawa (11/60). Based on these findings, it appears that *T. atripennis* is distributed across Japan, wherever *Onthophagus* species are found.

**Table 5:** Percentage of dung beetles associated with *Tokorhabditis atripennis* in each prefecture.

**Prefecture**	**% associated (no. associated site/no. examined)**						

	***Aphodius***	***Ataenius***	***Copris***	***Caccobius***	***Onthophagus***	***Phelotrupes***	**Total**
Hokkaido	0.00 (0/5)		0.00 (0/4)				0.00 (0/9)
Aomori	0.00 (0/17)						0.00 (0/17)
Iwate	0.00 (0/9)		0.00 (0/7)	1.79 (1/56)	0.00 (0/17)		1.12 (1/89)
Kanagawa					7.69 (3/39)	3.92 (3/51)	5.56 (5/90)
Gifu						0.00 (0/2)	0.00 (0/2)
Aichi	0.00 (0/6)			0.00 (0/17)	8.89 (4/45)	0.00 (0/13)	4.94 (4/81)
Kyoto						0.00 (0/2)	0.00 (0/2)
Nara	0.00 (0/62)			0.00 (0/11)	5.26 (5/95)	0.00 (0/13)	2.76 (5/181)
Miyazaki				12.5 (1/8)			12.5 (1/8)
Kumamoto	0.00 (0/4)		0.00 (0/14)	0.00 (0/10)	0.00 (0/5)	0.00 (0/2)	0.00 (0/35)
Kagoshima					0.00 (0/15)	0.00 (0/2)	0.00 (0/17)
Okinawa		0.00 (0/8)		0.00 (0/16)	18.3 (11/60)		13.1 (11/84)

Total	0.00 (0/103)	0.00 (0/8)	0.00 (0/25)	1.69 (2/118)	8.33 (23/276)	2.35 (2/85)	4.39 (27/615)

## Discussion

The six genera of dung beetles collected in this study can be categorized into two ecological types: tunneler beetles (*Copris*, *Caccobius*, *Onthophagus* and *Phelotrupes*), which dig tunnels beneath animal dung to create nests, and dweller beetles (*Aphodius* and *Ataenius*), which establish nests directly on the dung (Bornemissza, 1969; Bornemissza, 1976; Cambefort and Hanski, 1991). We only observed an association between the viviparous nematode *T. atripennis* and tunneler beetles, especially *Onthophagus* beetles (Table 3). Among *Onthophagus* beetles, the association rate was the highest for *O. apicetinctus*, which was primarily distributed in the Yaeyama Islands (Fig. 2; Tables 3 and 5). Conversely, *O. atripennis*, a species distributed throughout Japan, exhibited a higher association rate than the other species in different regions. This suggests that *T. atripennis* may reside in specific structures of the living environment of tunneler beetles, such as brood balls, and that specific biotic or abiotic factors in such environments may have driven the evolution of viviparity in nematodes.

Among the tunneler beetles, the association rates of *T. atripennis* with *O. apicetinctus* and *O. atripennis* were significantly higher than those with other dung beetles (Table 3). In addition, the phoretic association sites of *T. atripennis* appeared to vary between *Onthophagus* and other dung beetles (Table 4). This suggests that *T. atripennis* exhibits a preference for associating with *Onthophagus* beetles, showing a strong affinity for the two species, *O. apicetinctus* and *O. atripennis,* which could be considered the most suitable hosts for *T. atripennis*. The tendency of *T. atripennis* to attach to specific body parts of beetles suggests a symbiosis between *T. atripennis* and *Onthophagus* beetles. For example, dung beetles may act as carriers of nutrient-rich feces for *T. atripennis*, while *T. atripennis* could help create a favorable living environment for dung beetles.

Most nematodes described are oviparous; viviparous nematodes are considered rare. However, our study and previous studies have identified viviparous nematodes associated with *Onthophagus* dung beetles in different regions worldwide (Herrmann et al., 2013; Kanzaki et al., 2017, 2021; Ragsdale et al., 2022). Therefore, viviparous nematodes appear to have a broader distribution than previously thought. This suggests that the evolution of viviparity in nematodes may be related to the living environment of specific *Onthophagus* species. Further research is needed to clarify the ecological function and driving force of viviparity in relation to *Onthophagus* species. Since some researchers have reported nematode behavior in dung environments recreated in the laboratory (Kühne, 1996; Ledón-Rettig et al., 2018), these studies and our findings are expected to contribute to a better understanding of the life cycles of viviparous nematodes in natural environments and their interactions with other organisms.

## Figures and Tables

**Table S1:** Primer list used in this study.

Description	Primer sequence 5′-3′	Target	Reference
D2a	ACA AGT ACC GTG AGG GAA AGT TG	D2/D3 extension segments of the 28S ribosomal RNA	Nunn, 1992
D3b	TCG GAA GGA ACC AGC TAC TA		
D1F	AAG GAT TCC CTT AGT AAC GGC GAT TG	D1/D4 extension segments of the 28S ribosomal RNA	Kanzaki et al., 2021
D4R	GCG GTA TTT GCT ACT ACC AYY AMG ATC TG		
SSUF07	AAA GAT TAA GCC ATG CAT G	18S ribosomal RNA	Floyd et al., 2005
SSUR26	CAT TCT TGG CAA ATG CTT TCG		
18S-CL-F3	CTT GTC TCA AAG ATT AAG CCA TGC AT		Carta et al., 2018
18S-CL-R1	ACC TTG TTA CGA C T TTT G		

**Table S2:** Beetle number.

**Date**	**Name**	**Number**
2016.6.5	Onthophagus atripennis	10
2016.6.5	Onthophagus viduus	3
2016.6.5	Phelotrupes laevistriatus	1
2016.5.22	Phelotrupes laevistriatus	2
2016.7.5	Onthophagus atripennis	1
2016.11.15	Phelotrupes laevistriatus	1
2017.5.8	Onthophagus atripennis	1
2017.5.10	Onthophagus atripennis	1
2017.5.26	Onthophagus atripennis	5
2017.5.27	Phelotrupes laevistriatus	2
2017.5.27	Aphodius urostigma	2
2017.5.27	Aphodius rugosostriatus	2
2017.5.27	Aphodius botulus Balthasar	1
2017.5.27	Aphodius pusillus	3
2017.5.27	Aphodius superatratus	21
2017.5.27	Onthophagus ater	3
2017.5.27	Aphodius atratus	2
2017.5.27	Onthophagus ohbayashii	19
2017.5.27	Aphodius variabilis	1
2017.5.27	Aphodius sublimbatus	1
2017.5.27	Caccobius unicornis	1
2017.5.27	Onthophagus lenzii	2
2017.5.27	Aphodius uniplagiatus	1
2017.5.28	Phelotrupes laevistriatus	6
2017.5.28	Aphodius pusillus	1
2017.5.28	Aphodius superatratus	11
2017.5.28	Onthophagus ohbayashii	5
2017.5.28	Caccobius unicornis	2
2017.5.28	Onthophagus lenzii	2
2017.6.24	Onthophagus atripennis	1
2017.6.24	Onthophagus ater	9
2017.6.24	Onthophagus lenzii	1
2017.6.24	Aphodius japonicus	3
2017.6.24	Aphodius rectus	3
2017.6.24	Caccobius jessoensis	17
2017.6.24	Onthophagus nitidus	13
2017.7.2	Caccobius unicornis	5
2017.7.2	Caccobius brevis	3
2017.7.4	Onthophagus lenzii	15
2017.7.7	Onthophagus viduus	4
2017.7.7	Onthophagus apicetinctus	3
2017.8.28	Phelotrupes laevistriatus	2
2017.9.26	Caccobius jessoensis	5
2017.9.26	Onthophagus nitidus	1
2017.10.16	Phelotrupes auratus	2
2017.11.1	Aphodius botulus Balthasar	2
2017.11.1	Aphodius rectus	15
2018.5.11	Onthophagus ohbayashii	2
2018.5.11	Caccobius jessoensis	11
2018.7.24	Onthophagus atripennis	7
2018.7.24	Phelotrupes laevistriatus	5
2018.7.24	Aphodius pusillus	8
2018.7.24	Onthophagus ohbayashii	47
2018.7.24	Caccobius unicornis	8
2018.7.24	Onthophagus lenzii	6
2018.7.24	Aphodius rectus	1
2018.7.24	Onthophagus nitidus	4
2018.7.24	Aphodius uniformis	7
2018.8.22	Aphodius pusillus	3
2018.8.22	Aphodius uniformis	2
2018.8.28	Onthophagus nitidus	2
2018.8.29	Aphodius rugosostriatus	2
2018.8.29	Caccobius jessoensis	40
2018.8.29	Onthophagus nitidus	12
2018.8.29	Aphodius igai	2
2018.8.29	Copris acutidens	7
2018.8.29	Aphodius troitzkyi	5
2018.9.4	Aphodius urostigma	4
2018.9.4	Onthophagus lenzii	4
2018.9.4	Caccobius jessoensis	9
2018.9.4	Onthophagus nitidus	1
2018.9.4	Caccobius brevis	1
2018.9.4	Phelotrupes auratus	2
2018.9.4	Copris acutidens	14
2018.12.1	Caccobius unicornis	16
2018.12.1	Onthophagus murasakinus	1
2018.12.3	Onthophagus apicetinctus	4
2018.12.2	Onthophagus viduus	12
2018.12.2	Onthophagus apicetinctus	36
2020.11.16	Phelotrupes laevistriatus	2
2020.11.21	Phelotrupes laevistriatus	4
2020.11.23	Phelotrupes laevistriatus	2
2020.11.28	Phelotrupes laevistriatus	3
2021.5.14	Onthophagus atripennis	5
2021.5.14	Phelotrupes laevistriatus	10
2021.5.14	Onthophagus nitidus	4
2021.5.15	Onthophagus atripennis	2
2021.5.15	Phelotrupes laevistriatus	1
2021.5.16	Phelotrupes laevistriatus	1
2021.6.11	Phelotrupes laevistriatus	1
2021.6.11	Onthophagus fodiens	2
2021.6.14	Onthophagus nitidus	1
2021.6.25	Copris acutidens	4
2021.7.17	Ataenius australasiae	8
2021.8.2	Onthophagus atripennis	4
2021.8.2	Phelotrupes laevistriatus	9
2021.8.3	Phelotrupes laevistriatus	12
2021.8.3	Onthophagus nitidus	1
2021.8.4	Onthophagus atripennis	2
2021.8.4	Phelotrupes laevistriatus	9
2021.9.16	Phelotrupes laevistriatus	2
2021.9.30	Onthophagus atripennis	8
2021.9.30	Phelotrupes laevistriatus	1
2021.9.30	Onthophagus nitidus	2
2021.9.30	Onthophagus fodiens	1
2021.10.20	Phelotrupes laevistriatus	2
2021.10.20	Onthophagus ater	3
2021.10.21	Phelotrupes laevistriatus	3
2021.10.21	Onthophagus ater	4

**Table S3:** Summary of *Tokorhabditis atripennis* strains.

**Strain**	**Collection site**	**Accession No. LSU**	**Accession No. SSU**
KHA602	Chubu Univ, Kasugai	OR632650	OR632672
KHA603	Chubu Univ, Kasugai	OR632651	OR632673
KHA604	Chubu Univ, Kasugai	OR632652	OR632674
KHA605	Chubu Univ, Kasugai	OR632653	OR632675
KHA606	Kasugano Enchi, Nara	OR632654	OR632676
KHA607	Asajigahara, Nara	OR632655	OR632677
KHA608	Asajigahara, Nara	OR632656	OR632678
KHA612	Toino misaki, Kushima	OR632660	OR632682
KHA611	Ishigaki, Okinawa	OR632659	OR632682
KHA609	Ishigaki, Okinawa	OR632657	OR632679
KHA610	Ishigaki, Okinawa	OR632658	OR632680
KHA613	Morioka, Iwate	OR632661	OR632683
KHA614	Asajigahara, Nara	OR632662	OR632684
KHA615	Asajigahara, Nara	OR632663	OR632685
KHA616	Tonoshiro Park, Ishigaki	OR632664	OR632686
KHA619	Shinsakae Park, Ishigaki	OR632667	OR632689
KHA620	Tonoshiro Park, Ishigaki	OR632668	OR632690
KHA617	Tonoshiro Park, Ishigaki	OR632665	OR632687
KHA621	Tonoshiro Park, Ishigaki	OR632669	OR632691
KHA622	Tonoshiro Park, Ishigaki	OR632670	OR632692
KHA623	Tonoshiro Park, Ishigaki	OR632671	OR632693
KHA618	Tonoshiro Park, Ishigaki	OR632666	OR632688
SHR9	Meiji Univ, Kawasaki	OR681495	-
SHR12	Meiji Univ, Kawasaki	OR681496	-
SHR21	Meiji Univ, Kawasaki	OR681497	-
SHR24	Meiji Univ, Kawasaki	OR681498	OR717327
SHR16	Kurokawa, Kawasaki	OR681499	-
